# Restricted Kinematics in Children With Autism in the Execution of Complex Oscillatory Arm Movements

**DOI:** 10.3389/fnhum.2021.708969

**Published:** 2021-11-03

**Authors:** Zhong Zhao, Xiaobin Zhang, Haiming Tang, Xinyao Hu, Xingda Qu, Jianping Lu, Qiongling Peng

**Affiliations:** ^1^Institute of Human Factors and Ergonomics, College of Mechatronics and Control Engineering, Shenzhen University, Shenzhen, China; ^2^Shenzhen Guangming District Center for Disease Control and Prevention, Shenzhen, China; ^3^Department of Child Psychiatry of Shenzhen Kangning Hospital, Shenzhen Mental Health Center, Shenzhen, China; ^4^Developmental Behavioral Pediatric Department, Shenzhen Baoan Women’s and Children’s Hospital, Jinan University, Shenzhen, China

**Keywords:** autism, restrictedness and repetitive behavior, movement complexity, entropy, behavioral assessment

## Abstract

Restricted and repetitive behavior is a core symptom of autism spectrum disorder (ASD) characterized by features of restrictedness, repetition, rigidity, and invariance. Few studies have investigated how restrictedness is manifested in motor behavior. This study aimed to address this question by instructing participants to perform the utmost complex movement. Twenty children with ASD and 23 children with typical development (TD) performed one-dimensional, left-right arm oscillations by demonstrating varying amplitudes and frequencies. The entropy of amplitude and velocity was calculated as an index of kinematic complexity. Results showed that the velocity entropy, but not the amplitude entropy, was significantly lower in ASD than in TD (*p* < 0.01), suggesting restricted kinematics. Further analysis demonstrated that a significantly higher proportion of the velocity values was allocated at a low-speed level in the children with ASD (*p* < 0.01). A qualitative comparison of the complex movement with movement at preferred frequency suggested that the children with ASD might be less likely to shift away from the preferred movement. However, our study can be improved in terms of recruiting a larger sample of participants, measuring the level of motivation, and collecting both complex and preferred movements of the same participant.

## Introduction

Autism spectrum disorder (ASD) is a neurodevelopmental condition that greatly impairs the daily functionalities of affected individuals. According to the Diagnostic and Statistical Manual of Mental Disorders–5th Edition (DSM-V), restricted and repetitive behavior (RRB) is one of the two core symptoms of ASD (American Psychiatric and Association, 2013). People with autism spectrum disorder (ASD) exhibit a broad range of RRBs, such as repetitive motor activities and language, circumscribed interests, and adherence to specific routines (Kim and Lord, 2010).

The etiology of RRB remains unclear. It has been postulated that RRB might serve the purposes of reducing high arousal levels (Hutt et al., 1964; Repp et al., 1992) or deriving pleasure (Baron-Cohen, 1989; Klin et al., 2007). Notably, a sizable body of literature indicated that RRB could be the result of executive dysfunction (Turner, 1999; Lopez et al., 2005; D’Cruz et al., 2013). This hypothesis has received considerable support from studies that found a close relationship between the frontal lobe and RRB (Pierce and Courchesne, 2001; Shafritz et al., 2008), and between cognitive flexibility and RRB (Lopez et al., 2005; Tanimura et al., 2008). It was proposed that RRB may be derived from the inability to shift from preferred behaviors to new adaptive ones (Lopez et al., 2005; Corbett et al., 2009). People with ASD might be “locked into” a specific thought or behavior (Turner, 1999). Since RRB is an indispensable symptom of ASD, it is assumed that restricted kinematics (i.e., movement characterized by higher level of stereotypy, or lower degree of complexity) might be revealed in a wide variety of motor behaviors.

Despite motor deficits in people with ASD having been grossly documented, few studies have examined whether the restricted feature is manifested in motor behavior (Fournier et al., 2014; Li et al., 2019). The description of motor impairments in ASD could be dated back to the 1940s when Kanner reported the clumsy gait in children with ASD (Kanner, 1943). Many studies reported an association between ASD and abnormalities in postural control, gait patterns, and fine motor control (Esposito and Venuti, 2008; Fuentes et al., 2009; Fournier et al., 2014; Bojanek et al., 2020). Recently, a rigorous meta-analysis that compared the difference in motor coordination, arm movements, gait, and postural stability between individuals with ASD and those typically developing (TD) found that people with ASD present pronounced motor impairments across a wide range of behaviors (Fournier et al., 2010). However, whether and how the restricted feature is manifested in kinematics has been rarely reported.

Previous studies examining restricted kinematics in motor behavior have focused on the complexity of postural control and head movements in individuals with ASD (Fournier et al., 2014; Li et al., 2019; Zhao et al., 2021). For instance, Fournier et al. (2014) utilized multiscale entropy to quantify the complexity of postural control dynamics during quiet stance in children with ASD and age-matched TD children. Their results demonstrated that the children with ASD exhibited a reduced level of complexity both anteroposteriorly and mediolaterally, as observed in the center of pressure (COP) data (Fournier et al., 2014). Zhao et al. (2021) examined the level of head movement complexity in children with ASD during a face-to-face interaction and showed an elevated stereotypy in these children as compared with the TD peers. Noticeably, all these studies adopted unintentional tasks, in which participants were not instructed how to perform movements. It remains unexplored whether restricted kinematics could also be observed in individuals with ASD when they are deliberately instructed to perform the utmost complex movement.

The experimental design of this study was inspired by the research conducted by Słowiński et al. (2016), who investigated the existence of individual motor signature (IMS), a personalized motor feature that differentiates individuals. In the study of Słowiński et al. (2016), healthy participants were instructed to create one-dimensional, left-right oscillatory arm movement above a LeapMotion sensor. The movement of each participant was recorded three times, at least 1 week apart between two consecutive times. Although a complex movement was performed and the time series varied at different times, Słowiński et al. (2016) found that time-invariant and individual-specific kinematic properties were preserved in velocity distribution patterns, suggesting the existence of IMS. In light of this finding and given that ASD is characterized by stereotyped movement (Fournier et al., 2014; Li et al., 2019; Zhao et al., 2021), we were motivated to investigate whether restrictedness would be reflected in the movement of children with ASD when performing this particular motor task.

The significance of seeking restricted kinematics is of particular importance for obtaining objective behavioral markers of ASD. The current diagnosis of RRB in ASD heavily relies on the evaluation of an informant, which has been criticized as being laborious and having unreliable accuracy (Pyles et al., 1997). For example, the Autism Diagnostic Observation Schedule (ADOS) is considered a gold standard diagnostic instrument that requires significant clinical expertise. The length of the ADOS exam and shortage of trained clinicians significantly contributed to the delay in diagnosis (Shattuck et al., 2009). Data from the US reported that 13 months, on average, were required between the initial evaluation and confirmation of the diagnosis (Wiggins et al., 2006). Delayed diagnosis directly translates to postponed delivery of intervention programs, which negatively impacts the developmental outcomes of a child (Corsello, 2005). Therefore, a diagnostic tool that is both labor-saving and accurate is urgently called upon.

In this study, children with ASD and at least average non-verbal intellectual ability were recruited in order to ensure compliance with the experimental protocol. The instruction was to perform movements as complex as possible by demonstrating varying amplitudes and frequencies. Their movement was compared with that of TD participants.

We computed both the amplitude entropy and velocity entropy as indices of the kinematic complexity (the antonymous term for kinematic restrictedness). Entropy was originally introduced in the field of thermodynamics to denote the form of energy no longer available to do physical work (Clausius, 1867). It was later adapted to probability theory, information theory, and the theory of dynamical systems, where it is used to quantify the level of uncertainty, complexity, or irregularity (Shannon and Shannon, 1948; Kolmogorov, 1959). Entropy analysis has been previously performed to compute the complexity of movement in individuals with ASD, and a lower entropy value indicated a higher level of restrictedness/stereotypy (Fournier et al., 2014; Li et al., 2019; Zhao et al., 2021). In this study, we hypothesized that individuals with ASD would display more periodic movement patterns, and thus the entropy of amplitude and velocity would be significantly lower in ASD as compared with TD. In addition, we also examined the association between kinematic restrictedness and the RRB measured with the Repetitive Behavior Scale-Revised (RBS-R). A negative correlation between kinematic complexity and RBS-R scores was hypothesized.

## Materials and Methods

### Participants

The participants of this study came from a larger research project dedicated to seeking behavioral markers of ASD from social interaction (Zhao et al., 2021) and restricted behavior. The estimated sample size of this study was 34 based on data from a previous study that investigated behavioral complexity (Fournier et al., 2014) by implementing power analysis (between-group *t*-test, *d* = 1, α = 0.05, power = 0.8). Finally, 20 children with ASD and 23 children with TD were recruited, and the number of participants (*N* = 43 > 34) could provide sufficient power to register a statistical difference as the estimated sample. Children with ASD were recruited from the Department of Child Psychiatry in a first-class hospital. The diagnosis of ASD was confirmed with a series of rigorous procedures. The diagnosis was first made by a licensed psychiatrist with no less than 5 years of clinical experience by strictly following the DSM-IV criteria. Next, a senior psychiatrist further confirmed the diagnosis. A consultation with at least two additional senior psychiatrists would be involved if disagreement took place. The inclusion criteria were: (a) aged between 6 and 13 years old; (b) average non-verbal intellectual ability preselected by clinicians (IQ was subsequently tested by administering the Raven’s Advance Progress Matrices as IQ ≥ 70); and (c) absence of clinical comorbidities such as schizophrenia or attention deficit hyperactivity disorder (ADHD). The TD participants were recruited from local communities if no physical or mental disorders were reported. None of the TD participants declared the existence of ASD/ADHD in first-degree relatives. A written informed consent approved by the ethics committee of a local university was given by the caregivers of the participants. The experimental protocol conformed to the Declaration of Helsinki. Subject demographics are presented in Table 1.

**TABLE 1:** Subject demographics and group comparisons.

	**ASD**	**TD**	**Group comparison**	***p* value**
Sex (M:F)^†^	18:2	19:4	χ*^2^*(1) = 0.487	0.485
Age in months (Mean ± SD)^‡^	99 ± 24.6	110 ± 25.6	*t*(41) = 1.48	0.146
IQ (Mean ± SD)^‡^	102 ± 22.7	118 ± 15.5	*t*(32.9) = 2.75	0.012*

*^†^Chi-square test was performed.*

*^‡^Independent samples *t*-test was performed.*

*p < 0.05.

### Apparatus

**FIGURE 1:**
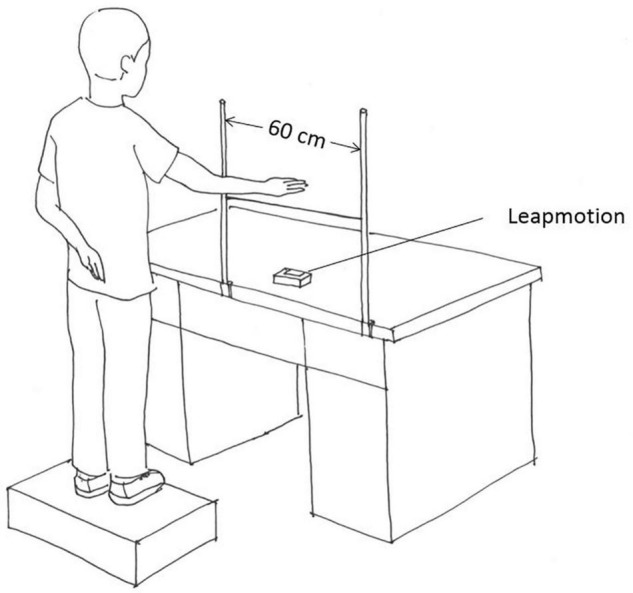
Schematic illustration of the experimental setup.

The experimental apparatus incorporated a computer (Lenovo Legion R720-15IKBN; Lenovo), a LeapMotion sensor (Leap Motion Inc.), two sticks, and a string (Figure 1). The LeapMotion sensor consists of two cameras and three infrared light-emitting diodes (LEDs, with a wavelength of 850 nm). It is a marker-less optical system that tracks the 3D movement of the palm and fingers of the users. Thanks to the low-cost and calibration-free characteristics of this device, this study utilized it to capture and save the spatial position of the center of the palm. The string was tied to the two sticks, and the distance between the sticks was 60 cm. Both the string and sticks allowed the participants to perform one-dimensional, left-right oscillatory movements. A solid box was offered to the participants to make sure that the dominant hand could move naturally and comfortably above the string.

### Repetitive Behavior Scale-Revised (RBS-R)

This study employed the Chinese version of RBS-R, which is a 43-item questionnaire evaluating the impression of a caregiver on the RRBs of children with ASD (Bodfish et al., 2000). The original RBS-R incorporated six subscales: Stereotyped Behavior, Self-Injurious Behavior, Compulsive Behavior, Ritualistic Behavior, Sameness Behavior, and Restricted Behavior. Caregivers were required to rate each item on a 4-point Likert scale, ranging from 0 (behavior does not occur) to 3 (behavior occurs and is a severe problem). In order to validate the inner structure of the measured RRBs, Lam and Aman (2007) performed factor analysis to achieve a five-factor solution, which could be summarized as “stereotypic behavior,” “self-injurious behavior,” “compulsive behavior,” “ritualistic/sameness behavior,” and “restricted interests” (Lam and Aman, 2007). In this study, the RBS-R scores were calculated based on the findings of Lam et al., and correlations between kinematic complexity and RBS-R total, as well as subscale scores, were computed.

### Experimental Procedure

The participants were required to perform continuous left-right oscillatory arm movements between the two sticks. The instruction was to perform a complex movements as possible. Prior to data collection, the experimenter behaviorally demonstrated that simple movement referred to periodic movement with mono amplitude and frequency, and complex movement represented unpredictable movement with varying amplitude and frequency. The participants had practice trials to assure that they fully understood the instructions. In order to avoid falsely registering the movement of the subdominant hand, the participants were told to keep the subdominant hand behind their back. Three trials of a movement task were recorded with each trial lasting 60 s. The participants were required not to withdraw their hand out of the recording zone or to put the subdominant hand in it. If any experimental rule was violated (e.g., withdrawing the hand from the recording zone, stopping the hand from moving voluntarily), the trial would be stopped and reinitiated. A break of 2–5 min was arranged between two consecutive trials to avoid confounding effects caused by fatigue.

### Data Analysis

**FIGURE 2:**
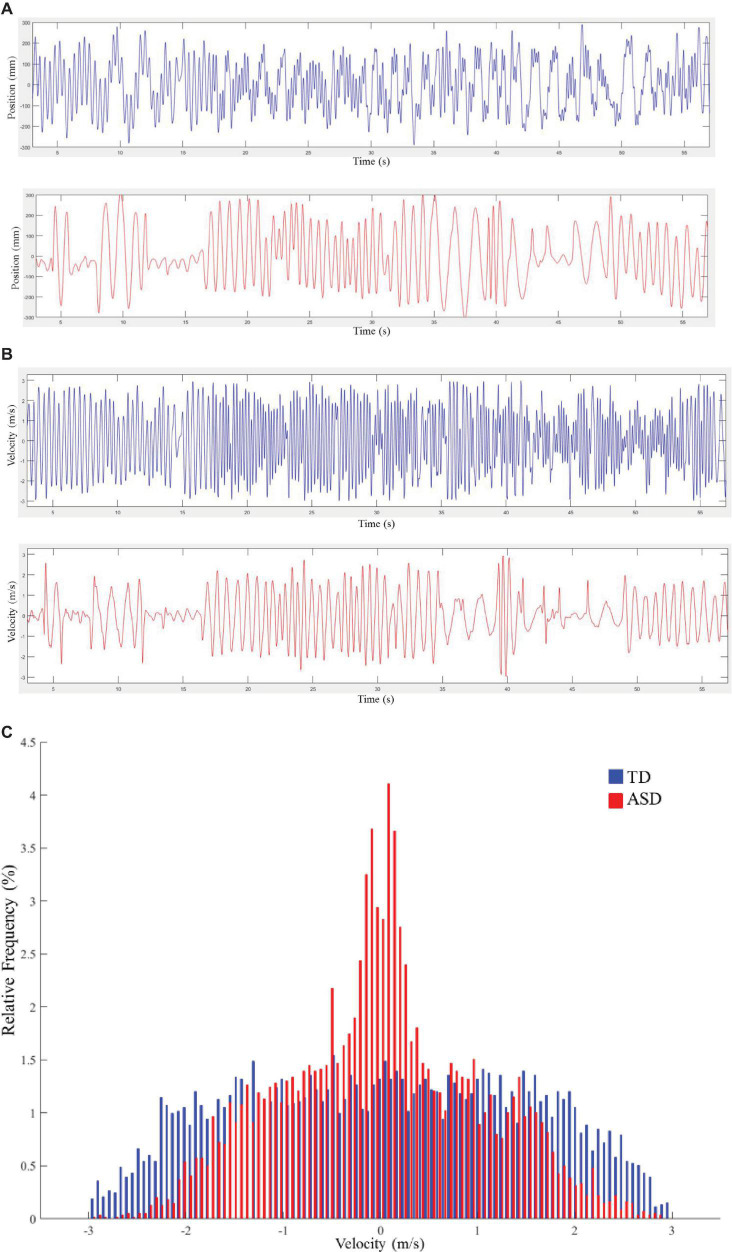
**(A)** Exemplary samples of time series for the position. Typical development (TD) is plotted in blue, and autism spectrum disorder (ASD) is plotted in red. **(B)** Exemplary samples of time series for velocity. TD is plotted in blue, and ASD is plotted in red. **(C)** Velocity distributions for typical movement trials from a child with TD and a child with ASD. TD is plotted in blue, and ASD is plotted in red. Each bin in the graph represents the proportion of corresponding velocity values in the trial.

The time series of the palm position was obtained as the raw data, which was further interpolated by means of the piecewise cubic Hermite interpolating polynomial method and filtered with a second-order low-pass Butterworth filter (5 Hz cut-off, Figure 2A). The first and the last 3 s were trimmed from the data analysis. Since the participants were required to perform complex oscillatory movements, each trial of movement was composed of multiple oscillations. This study focused on the difference in variation of oscillation amplitude and velocity between both groups of participants. The amplitude of a single oscillation was computed as the spatial distance between two consecutive endpoints (where velocity equaled 0). The set of amplitudes of a whole movement trial included the amplitude values of all single oscillations. Velocity (Figure 2B) was estimated by taking the first derivative of the position time series.

Before the calculation of entropy, we set the threshold range for amplitude and velocity values. Specifically, the threshold range for amplitude and velocity were set as 0–60 cm and -3 and 3 m/s, respectively. These threshold values were chosen empirically based on limits of the movement [e.g., the amplitude would not exceed 60 cm, since the maximum distance of the moving area was 60 cm, and it would be hard for the participants to move above 3 m/s in this experiment (Słowiński et al., 2016)]. Values out of these ranges were considered as noise, and they were discarded from further analysis. Subsequently, we used a normalized histogram with 101 equally distant bins (Słowiński et al., 2016) within the threshold range to compute the probability of each bin (Figure 2C illustrates two exemplary velocity distributions of a child with TD and a child with ASD), and calculated the Shannon entropy as:


$$\text{Entropy}=-\sum_{i=1}^{n}p\left(x_{i}\right)*\text{log}\_2p\left(x_{i}\right)$$

where *n* = 101; *p(x_*i*_)* = the probability of the *i*th bin.

### Statistical Analysis

This study implemented linear mixed-effects models (LMEMs) to investigate whether group membership was a significant predictor of kinematic complexity. The dependent variables were amplitude entropy and velocity entropy. We entered “Group” and “Participant’s sex” as fixed factors. The random factors were “Participants,” “Age,” “Participant’s IQ,” and “Order of the experimental trials.” The lme4 (Bates et al., 2015) package for R (R Core Team, 2013) was used to perform LMEMs, and the statistical significance of fixed factors was determined using Type II Wald chi-square tests in the “car” package (Fox et al., 2013). Spearman’s rank-order correlation tests were performed to compute correlations between kinematic complexity and RBS-R scores.

## Results

### Kinematic Complexity

**FIGURE 3:**
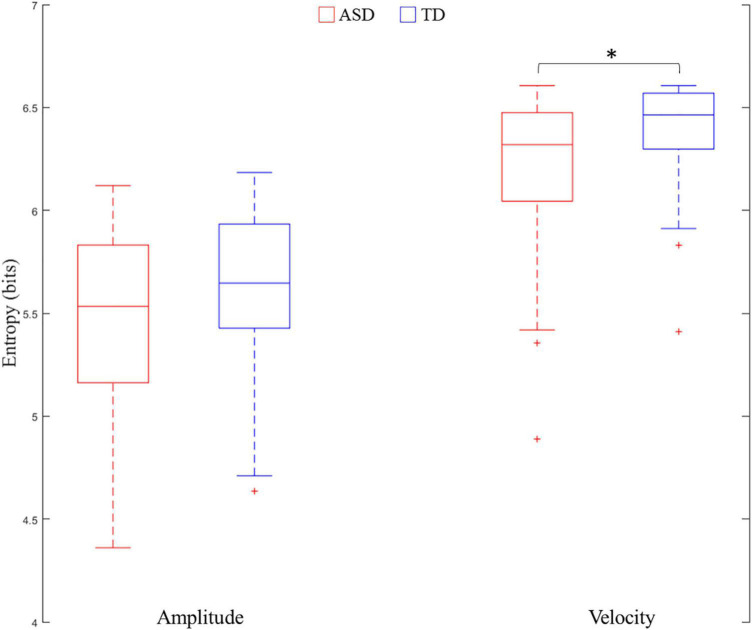
Comparisons of entropy of amplitude and velocity between ASD and TD. ^∗^ means *p* < 0.05.

The results demonstrated that Group was a significant predictor of velocity entropy [χ^2^(1) = 5.03, *p* = 0.02]. Velocity entropy in the ASD group (M ± SD: 5.94 ± 0.44) was lower than that in the TD group (M ± SD: 6.09 ± 0.32). However, the results failed to show that Group was a significant predictor of amplitude entropy [χ^2^(1) = 0.21, *p* = 0.65] (Figure 3). In both LMEMs, the sex of the participant was not found to be a significant predictor for either amplitude entropy [χ^2^(4) = 4.36, *p* = 0.36] or velocity entropy [χ^2^(4) = 8.08, *p* = 0.09].

**FIGURE 4:**
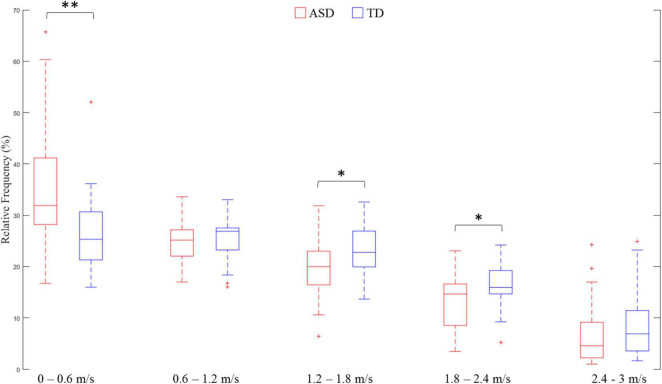
Comparison of velocity distribution in each absolute velocity segment. ^∗∗^ means *p* < 0.01; ^∗^ means *p* < 0.05.

Reduced velocity entropy indicated lower variance in velocity distribution in the ASD group as compared with the TD group. In order to investigate how velocity values were distributed for both groups of participants, we divided the absolute velocity^1^ into five equally divided segments between 0 and 3 m/s: Segment 1: 0–0.6 m/s; Segment 2:0.6–1.2 m/s; Segment 3: 1.2–1.8 m/s; Segment 4: 1.8–2.4 m/s; Segment 5: 2.4–3 m/s. The relative frequency of each segment (proportion of velocity values allocated in each segment) was calculated as the dependent variable. A two-way ANOVA was conducted with Group (ASD or TD) as the between-subjects factor, and Segment as the within-subjects factor. The results showed a Segment main effect [*F*(4,164) = 58.36, *p* < 0.01, $\eta_{p}^{2}$ = 0.587], and a Segment × Group interaction effect [*F*(4,164) = 5.14, *p* < 0.05, $\eta_{p}^{2}$ = 0.111]. Fisher’s least significant difference (LSD) tests demonstrated that the ASD group had a significantly greater proportion of velocity values allocated in Segment 1 (0–0.6 m/s) (*p* < 0.01), but significantly less proportions in Segment 3 (1.2–1.8 m/s) (*p* < 0.05) and Segment 4 (1.8–2.4 m/s) (*p* < 0.05) (Figure 4). These results demonstrated that the reduced velocity entropy in ASD was due to the fact that the participants with ASD performed more low-speed movements.

### Correlation Between Velocity Entropy and Repetitive Behavior Scale-Revised Scores

Spearman’s rank-order correlation tests failed to show that velocity entropy was significantly correlated to RBS-R subscale scores or the total score (all *p* > 0.05). These results indicated that velocity entropy might not be related to the RRBs of the children reported by caregivers.

## Discussion

This study investigated the existence of kinematic restrictedness in children with ASD and at least average non-verbal intelligence when performing volitional arm movements. The application of entropy analysis revealed a lower level of variance in the velocity distribution of participants with ASD, suggesting that children with ASD displayed a more restricted movement. Further analysis demonstrated that a higher proportion of velocity values was allocated at a low-speed level in children with ASD. Together with prior findings on restricted movement in individuals with ASD (Fournier et al., 2014; Li et al., 2019; Zhao et al., 2021), future studies on motor control in individuals with ASD are encouraged to examine the complexity of motor activity to complement conventional measures, such as the mean or peak of a given motor-related measure.

**FIGURE 5:**
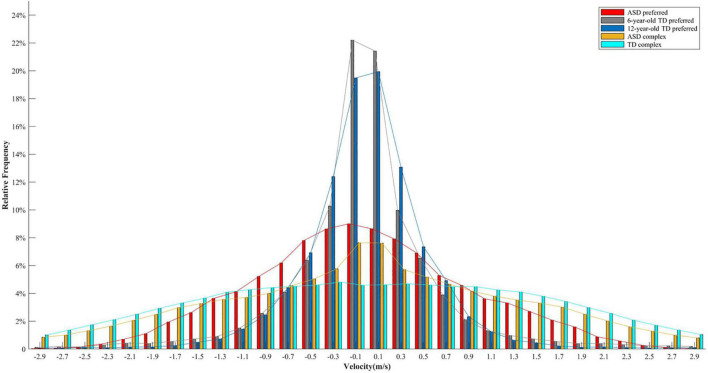
Velocity distributions for the preferred movement in 6-year-old children with TD, 12-year-old children with TD, and children with ASD, and for the complex movement in children with ASD and children with TD. Each distribution includes velocity values of all the participants in a specific group.

Why participants with ASD performed more low-speed movements? Previous studies demonstrated that individuals with ASD are less likely to shift away from their preferred response (Turner, 1999; Lopez et al., 2005; Corbett et al., 2009; D’Cruz et al., 2013). It was assumed that the greater proportion of low-speed movements might also be related to the incapacity of switching from one’s preferred movement patterns in children with ASD. In this study, the participants were instructed to perform continuous oscillatory movements. Abundant research investigating the dynamics of continuous oscillatory movements has evidenced the existence of preferred frequency (Kay et al., 1987; Temprado et al., 1999), which typically refers to the movement rate at which the performer feels most comfortable and the least energy cost is needed (Holt et al., 1995; Temprado et al., 1999). In order to save energy, the preferred frequency would not be too fast, and velocity distribution would be characterized by the majority of values allocated around zero. This idea could be confirmed by data from two other experiments measuring the preferred frequency of children with TD and ASD. The first experiment included ten 6-year-old and eight 12-year-old children with TD, whereas the second experiment included five children with ASD aged between 7 and 12 years (M ± SD: 116.4 ± 23.2 months). Children with TD were healthy participants enrolled from nearby communities and reported no physical or mental disorders. Children with ASD were high-functioning individuals recruited from Shenzhen Kangning Hospital. The objective of these two experiments was to assess eye-hand coordination, and preferred frequency was measured at the beginning of these two experiments, with the same experimental setup as this study. In both experiments, the experimenter behaviorally demonstrated that preferred frequency represented the tempo of movement that was neither too fast nor too slow as if one could do it all day long (Schmidt et al., 1993; Temprado et al., 1999; Zhao et al., 2020). The participants had practice trials to ensure that they fully understood the instruction. The preferred movement was recorded for 30 s in the first experiment (in the TD group), and for 60 s in the second experiment (in the ASD group). To make the lengths of these two experiments equivalent, the movement of the first 30 s in the second experiment was selected to perform further data analysis. As shown in Figure 5, the majority of velocity values was allocated at a low-speed level (around 0) in the preferred movement for both children with ASD and TD. In addition, the velocity distribution between the complex and the preferred movement in the children with ASD looked similar, but the difference in the TD group was much greater. In other words, Figure 5 qualitatively illustrates that the participants with ASD are less likely to shift from their preferred movement patterns. Strictly speaking, however, the velocity distribution of complex movements should be compared with that of the preferred movement of the same individual. Only in this way the deviation from the preferred movement could be precisely calculated. Future studies that will explore restricted kinematics are encouraged to record the preferred movement of participants as a baseline to confirm whether lower velocity entropy could be explained by the incapacity of switching from preferred movement in children with ASD.

### Limitations and Future Directions

The RBS-R scale was rated by caregivers to evaluate overt behavioral presentations of RRB in children with ASD. No significant correlation between velocity entropy and RBS-R scores seemed to indicate that velocity entropy was not related to the phenomenology of RRB. However, caution needs to be taken to make such a conclusion because of the fact that the sample size of this study was relatively small, and that Spearman’s correlation analysis is a conservative test to reveal a significant correlation between these two variables. A larger sample of participants with ASD could be tested in the future to further examine whether velocity entropy is associated with behavioral presentations of RRB reported by caregivers.

In our experimental design, it was hard to rule out the possibility that the lower velocity entropy was caused by a lack of motivation in the participants with ASD, as the motivation level of both groups of participants was not measured. However, the result that only velocity entropy, and not amplitude entropy, was lower in ASD as compared with TD indicated that the participants with ASD were equally motivated to change oscillation amplitude in order to perform a complex movement as possible. However, it is still strongly recommended that future studies incorporate motivation measures in the experiment to verify whether motivation plays a major role.

Our study included children with at least average non-verbal IQ to ensure the compliance of participants with ASD with the instructions. Since ASD displays significant heterogeneity (Jacob et al., 2019), whether our findings could be generalized to the whole ASD population requires further investigation. The DSM-V criteria classify patients with ASD into three severity levels depending on the condition of social communication and RRBs (American Psychiatric and Association, 2013). Future studies could be conducted to investigate whether kinematic restrictedness could predict the level of RRBs by examining individuals with ASD but with different severity. If this holds true, kinematic restrictedness could be potentially used to quantify RRBs in ASD.

This study used a LeapMotion sensor to collect data. As compared with other motion capture systems (e.g., Vicon and Optotrack), LeapMotion exhibits a greater advantage in terms of portability and cost (Shafritz et al., 2008). In addition, the contactless and calibration-free characteristics might make this instrument particularly useful for individuals with ASD, since greater difficulty would be experienced when instructing these people to wear devices/markers or perform calibrations. However, previous studies have reported inconsistent findings regarding the accuracy of the LeapMotion sensor (Weichert et al., 2013; Guna et al., 2014; Niechwiej-Szwedo et al., 2018; Fazeli and Peng, 2021). Some studies reported the high accuracy of this instrument in data collection (Weichert et al., 2013; Guna et al., 2014). For instance, Weichert et al. (2013) evaluated the accuracy and repeatability of the LeapMotion sensor in both static and dynamic tasks using an industrial robot with a reference pen. An average error of 0.2 mm in static tasks and 1.2 mm in dynamic tasks was reported for the LeapMotion sensor (Weichert et al., 2013). As a comparison, a greater error has been reported in other studies. For example, Niechwiej-Szwedo et al. (2018) examined the accuracy of the LeapMotion sensor during the performance of visually guided upper limb movements. Their results showed that the spatial and temporal errors were between 2–5 cm and 40 ± 44 ms, respectively. The average error in peak velocity was 0.024 ± 0.103 m/s (Niechwiej-Szwedo et al., 2018). In this study, our results showed that significant group differences existed across a wide range of the velocity spectrum ([0, 0.6] and [1.2, 2.4] m/s), which is much larger than the error in velocity reported by Niechwiej-Szwedo et al. (2018). Thus, it is assumed that the general pattern of our results might not be severely affected by the error of LeapMotion in data collection. However, strictly speaking, the accuracy of LeapMotion in our experiment should be validated by comparing it with other precise reference systems for motion capture. Future studies that will use LeapMotion in both research and practice might take into account the accuracy of this instrument.

The participants performed arm oscillations without having the hand constrained. In other words, their hands could move freely in different directions. Previous studies have mainly focused on investigating restricted kinematics in unintentional movements (Fournier et al., 2014; Li et al., 2019; Zhao et al., 2021). As the objective and the innovation of this study, we only explored restricted kinematics in volitional movements. In our experiment, the participants were particularly instructed to perform hand oscillations in the left-right direction. This suggests that the left-right direction captured the volitional movement and that movements on other dimensions could be viewed as unintentional. Thus, only movements in the left-right direction were analyzed in this study. All these relevant studies including ours suggest that restrictedness might widely exist in the kinematics of people with ASD. This information is useful for researchers to seek behavioral markers of ASD. Future studies might consider exploring restrictedness in various motor tasks with individuals with ASD.

## Conclusion

Consistent with the findings on restrictedness in postural control strategy and head movement (Fournier et al., 2014; Li et al., 2019; Zhao et al., 2021), our study demonstrated the existence of kinematic restrictedness in children with ASD when performing volitional complex arm movements. The exploration of restricted kinematics underlies the possibility of developing an objective and automatic assessment of RRBs in ASD. However, our study could be improved in terms of recruiting a larger sample of participants, measuring the level of motivation, and collecting both complex and preferred movements of the same participant.

## Data Availability Statement

The datasets presented in this article are not readily available because participants did not give consent to share data. Requests to access the datasets should be directed to the corresponding author.

## Ethics Statement

The studies involving human participants were reviewed and approved by the Institutional Review Board of Shenzhen University. Written informed consent to participate in this study was provided by the participants’ legal guardian/next of kin.

## Author Contributions

ZZ, XZ, and JL were involved in the experimental design and recruitment of participants. ZZ, HT, and XH analyzed the movement data and performed statistics. ZZ, XZ, XQ, and QP drafted and revised the manuscript. All authors contributed to the article and approved the submitted version.

## Conflict of Interest

The authors declare that the research was conducted in the absence of any commercial or financial relationships that could be construed as a potential conflict of interest.

## Publisher’s Note

All claims expressed in this article are solely those of the authors and do not necessarily represent those of their affiliated organizations, or those of the publisher, the editors and the reviewers. Any product that may be evaluated in this article, or claim that may be made by its manufacturer, is not guaranteed or endorsed by the publisher.

## Footnotes

1 Given the fact that the velocity distribution was approximately symmetry around 0 for all participants, we only used the absolute values.

## Abbreviations

ASD: autism spectrum disorder
DSM-V: the Diagnostic and Statistical Manual of Mental Disorders–5th Edition
IMS: individual motor signature
LMEM: linear mixed-effect model
RBS-R: Repetitive Behavior Scale-Revised
RRB: restricted and repetitive behavior
TD: typical development.
